# Aging & advocacy online: Disabled fashion bloggers and the pursuit of wellness.

**DOI:** 10.1093/geroni/igaf122.2323

**Published:** 2025-12-31

**Authors:** Allison Kabel, Kerri McBee-Black

**Affiliations:** Towson University, Towson, Maryland, United States; University of Missouri, Columbia, Missouri, United States

## Abstract

The goal of this study is to explore techniques used to advocate for social inclusion by fashion bloggers who are aging with disabilities and chronic health conditions. Online platforms have allowed people living with disabilities to work around establishment media barriers, share their perspectives, and educate the public, however, blog-based advocacy has been known to struggle with the required task of reaching its target audience, especially an audience that is not already committed to disability-related issues, such as accessibility in clothing, housing and transportation. The online forums discussed here include video logs, or ‘vlogs’, and weblogs, or ‘blogs’, associated with topics related to female disability-focused fashion. These data were publicly available social media platforms in accordance with ethical guidelines. Key findings/results included acknowledgement of the influential power of social media to reframe issues of aging and disability; Discovery of techniques for using fashion to raise awareness of social and policy issues, particularly those associated with older adult women and; Content creation as an advocacy tool for combating ageism and ableism. Implications for further research and education include the importance of working across disciplines, such as the social sciences, rehab health professions, design fields, education and communication professions, especially those specializing in social media.

